# Carlos Augusto Monteiro: nutrition and obesity

**DOI:** 10.2471/BLT.24.030824

**Published:** 2024-08-01

## Abstract

Carlos Monteiro talks to Gary Humphreys about Brazil’s dietary transition and the need for substantial change at every level of food production, marketing and consumption to address the global obesity pandemic.

*Q: You studied medicine. What drew you to specialize in nutrition and health?*

A: My original intent at medical school was to pursue community medicine. I completed a two-year residency in this field, working not in São Paulo city, but in its southern rural areas, particularly in a very underdeveloped region known as Vale do Ribeira. At the time, around 1972, there was no established primary health care there, and my fellow residents and I decided to set up a primary health-care programme which brought home to us the severe levels of under-nutrition in the region. It also provided some interesting insights into the way diet impacts health. For example, we noticed that small farmers growing diverse crops like beans and cassava in tea regions displayed far less severe under-nutrition than workers in tea and banana plantations because of their different diets. In response, we initiated a training programme for local teachers, and by equipping them with basic health and nutritional education were able to directly impact the community's health. This grassroots approach turned out to be quite successful and I sought to replicate the model in other regions. However, during the late 1970s, political turmoil under the military dictatorship restricted our movements, especially towards Central Brazil near the Amazon, which faced significant guerrilla activity. Consequently, I transitioned to an academic role in the nutrition department at the School of Public Health in São Paulo. So, initially at least, my engagement with nutrition was deeply rooted in practical, community-based encounters rather than academic study.

*Q: How did these insights translate into your academic research?*

A: They set me on the road I have followed ever since – pursuing the link between nutrition and health. My research focused on the nutritional transition in Brazil, documenting the dramatic shifts from undernutrition to obesity due to changes in diet, economic conditions and urbanization. The approach I took with colleagues was initially based on nutritional and epidemiological surveys, which indicated an alarming increase in obesity rates. For instance, starting in 2006, we observed about one million new cases of adult obesity per year. This realization prompted us to investigate dietary changes over time, particularly focusing on the increasing consumption of ultra-processed foods.

“We need a paradigm shift in how we view and consume food.”

*Q: What are ultra-processed foods?*

A: Ultra-processed foods are essentially manufactured products made from substances extracted from foods, like oils, fats, sugar, starch and proteins, or synthesized from food constituents, such as hydrogenated fats and modified starch. These foods are typically ready to eat or require minimal preparation and often contain additives like colours, flavours and preservatives. They also often contain higher levels of sugar, fat and salt, while being low in nutrients like fibre and vitamins. Such foods are often engineered to encourage overconsumption and are associated with higher calorie intake compared to traditional foods such as rice and beans.

*Q: How and why did you develop the NOVA classification and what is its significance for nutritional epidemiology?*

A: The NOVA classification categorizes foods based on the extent and purpose of their processing, rather than in terms of their nutrient constituents alone. The system breaks down foods into four groups: unprocessed or minimally processed foods, processed culinary ingredients, processed foods, and ultra-processed foods. The classification is designed to help understand how the processing level impacts health. Our studies have shown that higher consumption of ultra-processed foods is associated with higher risks of obesity, type 2 diabetes and cardiovascular diseases. 

*Q: How were you able to assess the extent to which people were consuming such foods?*

A: We utilized data from household purchase surveys, which are typically conducted to monitor inflation but also provide detailed information on food purchasing habits. These surveys helped us identify a shift from traditional staple foods like rice and beans to more ultra-processed products such as soft drinks, snacks and instant noodles. Over time, we incorporated more direct measures of food intake, such as 24-hour dietary recalls, which provide a more accurate picture of what people are actually consuming. These methods have been essential in tracking the transition and validating the health impacts of ultra-processed foods across different populations.

*Q: What drove the transition in the Brazilian diet?*

A: Broadly speaking, the transition tracked economic development, increased levels and distribution of income and urbanization. Between 1996 and 2006, despite slower economic growth compared to the so-called 'Brazilian Miracle' of the '70s, we saw significant advances in income distribution along with expansions in education, health services, and infrastructure like water and sewerage systems. Importantly, we saw that the nutritional status of the population was due not just to specific nutrition programmes but broader developmental policies that enhanced access to public services for the poor. For example, our studies indicated that improvements in maternal education were pivotal, accounting for about two thirds of the reduction in child stunting. This was followed by income growth, improving access to nutrition, better sanitation which among other things reduced the impact of waterborne diseases, and enhanced access to health care. These factors collectively showcased the multidimensional nature of nutritional status improvements, emphasizing that effective strategies extend beyond mere food supplementation to encompass broader socioeconomic policies. This was all good, of course, the positive side of economic development, but at the same time, as formerly marginalized communities were brought into the market, they began accessing different types of foods. While historically, individuals in those communities might have lacked sufficient access to any food, they now faced an abundance of cheap, readily available ultra-processed foods. And not just foods, of course; access to sugary soft drinks also increased, supported by aggressive marketing from the beverage industry. Ultra-processed foods, including soft drinks, are designed to be hyper-palatable and less satiating, leading people to consume more without feeling full. This shift is a crucial factor in understanding the rising obesity rates, notably around 2006 when we noticed an alarming increase in obesity rates linked to changes in diet, particularly an increase in ultra-processed food consumption. Other considerations are the decrease in physical activity associated with urbanization.

*Q: What are the implications of these findings for nutritional policy development?*

A: Our research underscores the importance of considering the types of foods available to consumers, not just the quantity. Effective public health strategies need to promote access to nutritious, unprocessed foods while curbing the prevalence and availability of ultra-processed products. But effective public policy should also address the multisectoral nature of nutrition and health, encompassing education, income distribution, and access to health services. By understanding and intervening in these interconnected areas, we can better combat nutritional challenges and improve overall public health outcomes.

*Q: How have your findings influenced dietary guidelines and public health policies in Brazil?*

A: Our research significantly influenced the development of Brazilian dietary guidelines, notably those issued in 2014. These guidelines emphasize the health risks associated with ultra-processed foods and recommend minimizing their consumption. This approach has been adopted in numerous public health initiatives across Brazil, impacting millions of people by integrating these guidelines into national health protocols for managing conditions like diabetes and hypertension. The guidelines are designed to connect with people, presenting in simple-to-understand terms and using photos of typical meals – breakfasts, lunches, dinners that align with our guidelines. This visual approach helps people understand the recommendations, even if they can't read. We also recommend fruit consumption in its whole form rather than juice. For those who do drink juice, we suggest making it fresh at home, while recognizing that this isn't always feasible daily due to the time, cost, and effort involved. We also emphasize the importance of traditional diets such as rice and beans and promote drinking water over any other beverage. We are keen to promote and protect traditional dietary and culinary practices, which are still an important component of the nutritional landscape in Brazil as they are in many lower-income countries. To some extent we are defending something which still exists rather than trying to establish something that has been destroyed, as is the case in many high-income countries, where ultra-processed convenience foods are prevalent, and shifting back to traditional diets is much more challenging.

*Q: Worldwide adult obesity has more than doubled since 1990, and adolescent obesity has quadrupled. What can be done to reverse this trend?*

A: The challenge remains in translating the growing body of evidence into effective public policy that can curb the rise in consumption of ultra-processed foods. By continuing to refine our understanding and communication of these issues, we can better guide public health interventions and promote healthier dietary patterns globally. Clearly, we need to do a better job of communicating but there are other levers we can pull. In Brazil, the authorities are currently discussing tax reforms that would apply zero tax to unprocessed and minimally processed foods, while higher taxes might be applied to ultra-processed foods and sugary drinks. This is directly influenced by our dietary guidelines. But beyond dietary guidelines, we need comprehensive food system reforms. This includes promoting ‘real’, unprocessed foods and reducing the availability and appeal of ultra-processed options. As things stand, a lot of responsibility is placed on the consumer, the individual, but actually the environment needs to change. It is analogous to emphasizing the recycling of plastic rather reducing its use in packaging and single-use applications. With nutrition, long term change will require substantial changes at every level of food production, marketing and consumption.

*Q: Can appetite-suppressing drugs such as semaglutide make a difference?*

A: I don’t believe so. While they may offer immediate – albeit transient – relief from over-consumption they don't address the root cause: the food industrial complex itself, the food system. Also, it remains to be seen what kind of side effects and adverse events are going to occur. No, the truth is we need a paradigm shift in how we view and consume food, focusing more on life-sustaining nutrition rather than short-term fixes.

